# Children with hyperactive-impulsive disorder benefit from yoga training

**DOI:** 10.1192/j.eurpsy.2021.574

**Published:** 2021-08-13

**Authors:** N. Kiseleva, S. Kiselev

**Affiliations:** 1 Laboratory For Brain And Neurocognitive Development, Ural Federal University, Ekaterinburg, Russian Federation; 2 Clinical Psychology, Ural Federal University, Ekaterinburg, Russian Federation

**Keywords:** hyperactive-impulsive disorder, executive abilities, yoga training

## Abstract

**Introduction:**

It is known that children with hyperactive-impulsive disorder have deficit in executive abilities. It is important to search for effective approaches for developing executive abilities in children with this disorder.

**Objectives:**

The goal of this study was to reveal effect of yoga training on executive abilities in 8-9 years of age children with hyperactive-impulsive disorder. We compared the efficacy of two methods of training (yoga training vs. conventional motor exercises) in a randomized controlled pilot study.

**Methods:**

18 children with hyperactive-impulsive disorder at the age of 8-9 years were included and randomly assigned to treatment conditions according to a 2×2 crossover design. Children from intervention group participated in 12 weeks of yoga training that included body-oriented activity and breathing exercises. To assess the executive functions we used 3 subtests from NEPSY (Auditory Attention and Response Set, Visual Attention, Statue). Effects of training were analyzed by means of an ANOVA for repeated measurements.

**Results:**

The ANOVA has revealed (p<.05) that for all used subtests (Auditory Attention and Response Set, Visual Attention, Statue) the yoga training was superior to the conventional motor training, with effect sizes in the medium-to-high range (0.43-0.88).

**Conclusions:**

The findings from this pilot study suggest that yoga training have positive effect on executive abilities in children with hyperactive-impulsive disorder. It influences predominantly the selective and sustained attention, inhibition, monitoring, and self-regulation. However, it is necessary to do further research into the impact of yoga exercises on the prevention and treatment of hyperactive-impulsive disorder in children.

